# Applications of Natural Language Processing in Biodiversity Science

**DOI:** 10.1155/2012/391574

**Published:** 2012-05-22

**Authors:** Anne E. Thessen, Hong Cui, Dmitry Mozzherin

**Affiliations:** ^1^Center for Library and Informatics, Marine Biological Laboratory, 7 MBL Street, Woods Hole, MA 02543, USA; ^2^School of Information Resources and Library Science, University of Arizona, Tucson, AZ 85719, USA

## Abstract

Centuries of biological knowledge are contained in the massive body of scientific literature, written for human-readability but too big for any one person to consume. Large-scale mining of information from the literature is necessary if biology is to transform into a data-driven science. A computer can handle the volume but cannot make sense of the language. This paper reviews and discusses the use of natural language processing (NLP) and machine-learning algorithms to extract information from systematic literature. NLP algorithms have been used for decades, but require special development for application in the biological realm due to the special nature of the language. Many tools exist for biological information extraction (cellular processes, taxonomic names, and morphological characters), but none have been applied life wide and most still require testing and development. Progress has been made in developing algorithms for automated annotation of taxonomic text, identification of taxonomic names in text, and extraction of morphological character information from taxonomic descriptions. This manuscript will briefly discuss the key steps in applying information extraction tools to enhance biodiversity science.

## 1. Introduction

 Biologists are expected to answer large-scale questions that address processes occurring across broad spatial and temporal scales, such as the effects of climate change on species [1, 2]. This motivates the development of a new type of data-driven discovery focusing on scientific insights and hypothesis generation through the novel management and analysis of preexisting data [3, 4]. Data-driven discovery presumes that a large, virtual pool of data will emerge across a wide spectrum of the life sciences, matching that already in place for the molecular sciences. It is argued that the availability of such a pool will allow biodiversity science to join the other “Big” (i.e., data-centric) sciences such as astronomy and high-energy particle physics [5]. Managing large amounts of heterogeneous data for this Big New Biology will require a cyberinfrastructure that organizes an open pool of biological data [6].

**Figure 1:**
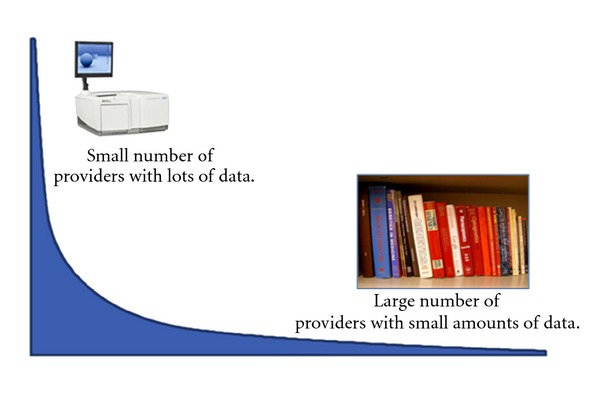
The long tail of biology. Data quantity, digitization, and openness can be described using a hyperbolic (hollow) curve with a small number of providers providing large quantities of data, and a large number of individuals providing small quantities of data.

To assess the resources needed to establish the cyberinfrastructure for biology, it is necessary to understand the nature of biological data [4]. To become a part of the cyberinfrastructure, data must be ready to enter a digital data pool. This means data must be digital, normalized, and standardized [4]. Biological data sets are heterogeneous in format, size, degree of digitization, and openness [4, 7, 8]. The distribution of data packages in biology can be represented as a hollow curve [7] (Figure 1). To the left of the curve are the few providers producing large amounts of data, often derived from instruments and born digital such as in molecular biology. To the right of the curve are the many providers producing small amounts of data. It is estimated that 80% of scientific output comes from these small providers [7]. Generally called “small science,” these data are rarely preserved [9, 10]. Scientific publication, a narrative explanation derived from primary data, is often the only lasting record of this work.

The complete body of research literature is a major container for much of our knowledge about the natural world and represents centuries of investment. The value of this information is high as it reflects observations that are difficult to replace if they are replaceable at all [7]. Much of the information has high relevance today, such as records on the historical occurrence of species that will help us better understand shifting abundances and distributions. Similarly, taxonomy, with its need to respect all nomenclatural acts back to the 1750s, needs to have access to information contained exclusively within this body of literature. Unfortunately, this knowledge has been presented in the narrative prose such that careful reading and annotation are required to make use of any information [11] and only a subset has been migrated into digital form.

The number of pages of the historical biodiversity literature is estimated to be approximately hundreds of millions [12]. Currently, over 33 million pages of legacy biology text are scanned and made available online through the Biodiversity Heritage Library (http://www.biodiversitylibrary.org/) and thousands of new digital pages are published every month in open-access biology journals (estimated based on 216 journals publishing approx 10 articles per month of less than 10 pages; http://www.doaj.org/doaj?cpid=67&func=subject). Additional biologically focused digital literature repositories can be found here (http://www.library.illinois.edu/nhx/resources/digitalresourcecatalogs.html).

The information is in human-readable form but is too much for a person to transform into a digital data pool. Machines can better handle the volume, but cannot determine which elements of the text have value. In order to mobilize the valuable content in the literature, we need innovative algorithms to translate the entirety of the biological literature into a machine-readable form, extract the information with value, and feed it in a standards-compliant form into an open data pool. This paper discusses the application of natural language processing algorithms to biodiversity science to enable data-driven discovery.

## 2. Overview

### 2.1. Information Extraction

Research addressing the transformation of natural language text into a digital data pool is generally labeled as “information extraction” (IE). An IE task typically involves a corpus of source text documents to be acted upon by the IE algorithm and an extraction template that describes what will be extracted. For a plant character IE task, (e.g., [13]), a template may consist of taxon name, leaf shape, leaf size, leaf arrangement, and so forth (Table 1). The characteristics of the source documents and the complexity of the template determine the difficulty level of an IE task. More complex IE tasks are often broken down to a series (stacks) of sub tasks, with a later subtask often relying on the success of an earlier one.  Table 2 illustrates typical subtasks involved in an IE task. Note, not all IE tasks involve all of these subtasks. Examples of information extraction tools for biology (not including biodiversity science) can be found in Table 3.

The IE field has made rapid progress since the 1980s with the Message Understanding Conferences (MUCs) and has become very active since the 1990s due largely to the development of the World Wide Web. This has made available huge amounts of textual documents and human-prepared datasets (e.g., categorized web pages, databases) in an electronic format. Both can readily be used to evaluate the performance of an IE system. The massive production of digital information demands more efficient, computer-aided approaches to process, organize, and access the information. The urgent need to extract interesting information from large amounts of text to support knowledge discovery was recognized as an application for IE tools (e.g., identifying possible terrorists or terrorism attacks by extracting information from a large amount of email messages). For this reason, IE and other related research have acquired another, more general label “text data mining” (or simply “text mining”).

Information extraction algorithms are regularly evaluated based on three metrics: recall, precision, and the F score. Consider an algorithm trained to extract names of species from documents being run against a document containing the words: cat, dog, chicken, horse, goat, and cow. The recall would be the ratio of the number of “species words” extracted to the number in the document (6). So, an algorithm that only recognized cat and dog would have low recall (33%). Precision is the percentage of what the algorithm extracts that is correct. Since both cat and dog are species words, the precision of our algorithm would be 100% despite having a low recall. If the algorithm extracted all of the species words from the document, it would have both high precision and recall, but if it also extracts other words that are not species, then it would have low precision and high recall. The *F* score is an overall metric calculated from precision and recall when precision and recall are considered equally important:

(1) $$F\;\text{score}=2\left(\frac{\left(\text{precision}\;*\;\text{recall}\right)}{\left(\text{precision}+\text{recall}\right)}\right).$$

**Figure 2:**
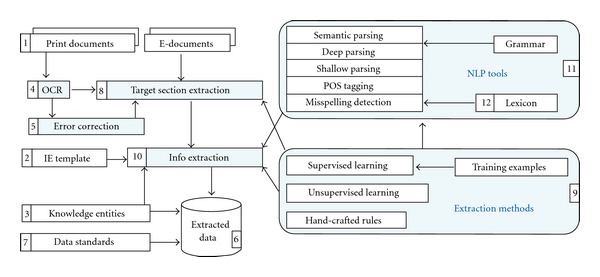
A reference system architecture for an example IE system. Numbers correspond to the text.

Before we review current IE systems for biodiversity science, we will first present a reference system architecture for a model IE system that covers the entire process of an IE application (Figure 2). In reviewing variant systems, we will refer to this reference architecture.

The blue-shaded areas in Figure 2 illustrate an IE system. The inputs to the IE system include source documents in a digital format (element number 1 in Figure 2), an IE template which describes the IE task (2) and knowledge entities to perform the task (3). If documents are not in a digital format, OCR technologies can be used to make the transition (4; see below section on digitization), but then it is necessary to correct OCR errors before use (5). In this model system, we use “IE template” to refer not only to those that are well defined such as the leaf character template example in Table 1, but also those more loosely defined. For example, we also consider lists of names and characters to be IE templates so the reference system can cover Named Entity Recognition systems (see below for examples) and character annotation systems (see below for examples). Knowledge entities include, for example, dictionaries, glossaries, gazetteers, or ontologies (3). The output of an IE system is often data in a structured format, illustrated as a database in the diagram (6). Ideally the structured format conforms to one of many data standards (7), which can range from relational database schemas to RDF. The arrow from Knowledge Entities to Extracted Data illustrates that, in some cases, the extracted data can be better interpreted with the support of knowledge entities (like annotation projects such as phenoscape, http://phenoscape.org/wiki/Main_Page). The arrow from Data Standards to Extracted Data suggests the same.

NLP techniques are often used in combination with extraction methods (including hand-crafted rules and/or machine learning methods). Often the input documents contain text that are not relevant to an IE task [14]. In these cases, the blocks of text that contain extraction targets need to be identified and extracted first to avoid the waste of computational resources (8). An IE method is often used for this purpose (9). From the selected text, a series of tasks may be performed to extract target information (10) and produce final output (6; see also IE subtasks in Table 2). This is often accomplished by first applying NLP techniques (11) and then using one or a combination of extraction methods (9). The arrow from extraction methods to NLP tools in Figure 2 indicates that machine learning and hand-crafted rules can be used to adapt/improve NLP tools for an IE task by, for example, extracting domain terms to extend the lexicon (12) used by a syntactic parser or even create a special purpose parser [15]. One important element that is not included in the model (Figure 2) is the human curation component. This is important for expert confirmation that extraction results are correct.

### 2.2. Natural Language Processing

IE is an area of application of natural language processing (NLP). NLP enables a computer to read (and possibly “understand”) information from natural language texts such as publications. NLP consists of a stack of techniques of increasing sophistication to progressively interpret language, starting with words, progressing to sentence structure (syntax or syntactic parsing), and ending at sentence meaning (semantics or semantic parsing) and meaning within sequences of sentences (discourse analysis). Typically an NLP technique higher in the stack (discourse analysis) utilizes the techniques below it (syntactic parsing). A variety of NLP techniques have been used in IE applications, but most only progress to syntactic parsing (some special IE applications specifically mentioned in this paper may not use any of the techniques). More sophisticated techniques higher in the stack (semantic parsing and discourse analysis) are rarely used in IE applications because they are highly specialized that is, cannot be reliably applied in general applications and are more computationally expensive.

**Figure 3:**
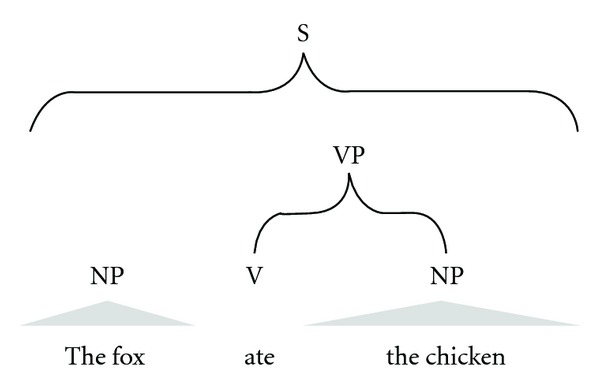
An example of shallow parsing. Words and a sentence (S) are recognized. Then, the sentence is parsed into noun phrases (NP), verbs (V), and verb phrases (VP).

**Figure 4:**
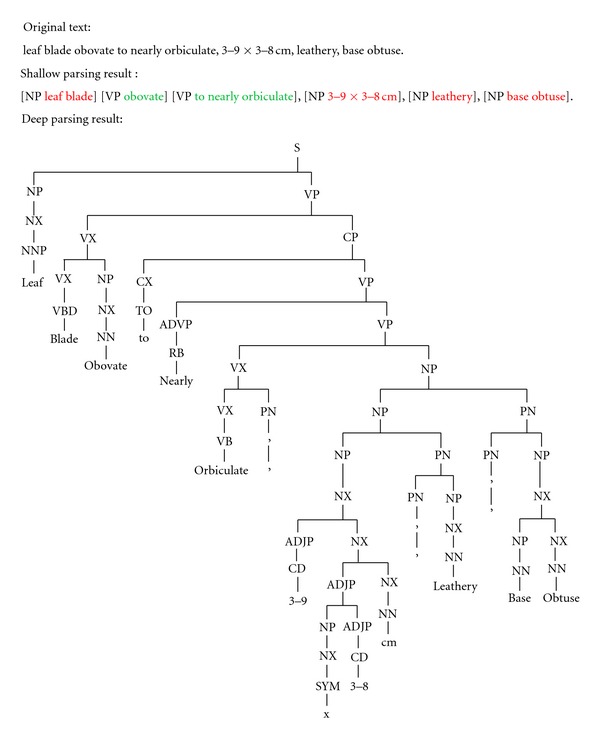
Shallow-vs-Deep-Parsing. The shallow parsing result produced by GENIA Tagger (http://text0.mib.man.ac.uk/software/geniatagger/). The deep parsing result produced by Enju Parser for Biomedical Domain (http://www-tsujii.is.s.u-tokyo.ac.jp/enju/demo.html). GENIA Tagger and Enju Parser are products of the Tsujii Laboratory of the University of Tokyo and optimized for biomedical domain. Both Parsing results contain errors, for example “obovate” should be an ADJP (adjective phrase), but GENIA Tagger chunked it as a VP (verb phrase). “blade” is a noun, but Enju parser parsed it as a verb (VBD). This is not to criticize the tools, but to point out language differences in different domains could have a significant impact on the performance of NLP tools. Parsers trained for a general domain produce erroneous results on morphological descriptions [16].

Syntactic parsing can be shallow or deep. Shallow syntactic parsing (also called “chunking”) typically identifies noun, verb, preposition phrases, and so forth in a sentence (Figure 3), while deep syntactic parsing produces full parse trees, in which the syntactic function (e.g., Part of Speech, or POS) of each word or phrase is tagged with a short label (Figure 4). The most commonly used set of POS tags used is the Penn Treebank Tag Set (http://bulba.sdsu.edu/jeanette/thesis/PennTags.html), which has labels for different parts of speech such as adjective phrases (ADJP), plural nouns (NNP), and so forth. Not all shallow parsers identify the same set of phrases. GENIA Tagger, for example, identifies adjective phrases (ADJP), adverb phrases (ADVP), conjunctive phrases (CONJP), interjections (INTJ), list markers (LST), noun phrases (NP), prepositional phrases (PP), participles (PRT), subordinate clauses (SBAR), and verb phrases (VP). Some shallow parsing tools are the Illinois Shallow Parser (http://cogcomp.cs.illinois.edu/page/software_view/13) the Apache OpenNLP (http://incubator.apache.org/opennlp/index.html), and GENIA Tagger (http://www-tsujii.is.s.u-tokyo.ac.jp/GENIA/tagger/). Deep parsing tools include Stanford Parser (http://nlp.stanford.edu/software/lex-parser.shtml), Link Parser (http://www.link.cs.cmu.edu/link/) and Enju Parser (http://www-tsujii.is.s.u-tokyo.ac.jp/enju/). A majority of IE applications use the shallow parsing technique, but the use of deep parsing techniques is on the rise in biology applications. This is driven in part because shallow parsing is not adequate to extract information from biology text [27–29].

Several NLP approaches are available for IE applications in biology that go beyond shallow parsing and are not mutually exclusive.

1. *Pattern matching* approaches exploit basic patterns in text to extract information. An example pattern is “enzyme activates protein” or X activates Y. The computer would look for the specific text pattern and assume that all X are enzymes or all Y are proteins. Dictionary-based IE is a variant of pattern matching that focuses on finding words in text that are contained in a dictionary previously given to the computer. For example, the computer might be given a list of enzyme names (such as the UM-BBD list of enzyme names, http://umbbd.msi.umn.edu/servlets/pageservlet?ptype=allenzymes; X in previous example). Once the enzyme name is located, the computer can infer the pattern that it “activates Y.” Another variant of pattern matching is the preposition-based parsing which focuses on finding prepositions like “by” and “of” and filling a basic template with information surrounding that preposition. An example of this would be “Y is activated by X.” Pattern matching suffers from the difficulty in accounting for the wide array of linguistic patterns used in text (X activates Y, Y is activated by X, Y was activated by X, X activated Y, Y is activated via X, X, which activates Y, etc.). Many of these systems extract phrases or sentences instead of structured facts, which limits their usefulness for further informatics. An example system that uses pattern matching is given in Krauthammer et al. [37].
2. *Full parsing* approaches expand on shallow parsing to include an analysis of sentence structure (i.e., syntax, see Figure 3). The biggest challenge with this approach is the special language of biology-specific texts. Most existing full-parsing systems are designed to handle general language texts, like news articles. The approach is also limited by grammar mistakes in the literature, which are often due to nonnative English speakers. Full parsing often runs into ambiguity due to the many ways a sentence (even moderately complex) can be interpreted by a machine. Sentence fragments, such as titles or captions, can also cause problems. UniGene Tabulator is an example of a full parser for biology [38].
3. *Probability-based* approaches offer a solution to the linguistic variability that confounds full parsing. These approaches use weighted grammar rules to decrease sensitivity to variation. The weights are assigned through processing of a large body of manually annotated text. Probabilistic grammars are used to estimate the probability that a particular parse tree will be correct or the probability that a sentence or sentence fragment has been recognized correctly. Results can be ranked according to the probabilities. Nasr and Rambow give an example of a probability-based parser [39].
4. *Mixed syntactic-semantic* approaches take advantage of syntactic and semantic knowledge together. This essentially combines part-of-speech taggers with named-entity recognition, such as in the BANNER system [40]. This removes reliance on lexicons and templates. This approach will be discussed further below.
5. *Sub language*-*driven* approaches use the specialized language of a specific community. A specialized sub language typically has a set of constraints that determine vocabulary, composition, and syntax that can be translated into a set of rules for an algorithm. Algorithms for use in processing biology text must cope with specialized language and the telegraphic sentence structure found in many taxonomic works. Being unaware of a sub language will often lead to incorrect assumptions about use of the language. Metexa is an example of a tool that uses a specialized sub language in the radiology domain [41].

NLP techniques are often used as a (standard) initial text processing procedure in an IE application. Once a computer has an understanding of the syntactic and/or semantic meaning of the text, other methods, such as manually derived rules or machine learning based methods, are then often used for further information extraction.

### 2.3. Machine Learning

Machine learning has been used in IE applications since 1990s. It is a process by which a machine (i.e., computer algorithm) improves its performance automatically with experience [42]. Creating extraction rules automatically by machine learning are favored over creating them manually because the hand-crafted rules take longer to create and this time accumulates for each new document collection [43]. As a generic method, machine-learning applications may be found in all aspects of an IE system, ranging from learning lexicons for a syntactic parser, classifying and relating potential extraction targets, to fitting extracted entities into an extraction template.

Learning can take various forms including rule sets, decision trees, clustering algorithms, linear models, Bayesian networks, artificial neural networks, and genetic algorithms (which are capable of mimicking chromosome mutations). Some machine-learning algorithms (e.g., most classification algorithms such as decision trees, naïve Baysian, Support Vector Machines) rely on substantial “training” before they can perform a task independently. These algorithms fall in the category of “supervised machine learning.” Some other algorithms (e.g., most clustering algorithms) require little or no training at all, so they belong to the “unsupervised machine learning” category. Due to the considerable cost associated with preparing training examples, one research theme in machine learning is to investigate innovative ways to reduce the amount of training examples required by supervised learning algorithms to achieve the desired level of performance. This gave rise to a third category of machine learning algorithms, “semisupervised.” Co-training is one of the learning approaches that falls into this category. Co-training refers to two algorithms that are applied to the same task, but learn about that task in two different ways. For example, an algorithm can learn about the contents of a web site by (1) reading the text of the web site or (2) reading the text of the links to the web site. The two bodies of text are different, but refer to the same thing (i.e., the web site). Two different algorithms can be used to learn about the web site, feed each other machine-made training examples (which reduces the requirements of human-made training examples), and often make each other better. However, co-training requires two independent views of the same learning task and two independent learners. Not all learning tasks fulfill these requirements. One line of research in NLP that uses co-training is word sense disambiguation [44]. We are not aware of the use of this learning approach in biodiversity information extraction. The best learning algorithm for a certain task is determined by the nature of the task and characteristics of source data/document collection, so it is not always possible to design an unsupervised or semisupervised algorithm for a learning task (i.e., an unsupervised algorithm to recognize human handwriting may not be possible).

The form of training examples required by a supervised algorithm is determined largely by the learning task and the algorithm used. For example, in Tang and Heidorn [13], the algorithm was to learn (automatically generate) rules to extract leaf properties from plant descriptions. A training example used in their research as well as the manually derived, correct extraction is in Box 1 (examples slightly modified for human readability).

By comparing original text (italics) and the text in bold, the algorithm can derive a set of candidate extraction rules based on context. The algorithm would also decide the order that the extraction rules may be applied according to the rules' reliability as measured with training examples. The more reliable rules would be utilized first. Two extraction rules generated by the Tang and Heidorn [13] algorithm are shown in Box 2. Rule  1 extracts from the original text any leaf shape term (represented by <leafShape>) following a term describing leaf blade (represented by <PartBlade>) and followed by a comma (,) as the leafShape (represented by the placeholder $1). Rule  2 extracts any expression consisting of a range and length unit (represented by <Range><LengUnit>) that follows a comma (,) and is followed by another comma (,) and a leaf base term (represented by <PartBase>) as the bladeDimension.

These rules can then be used to extract information from new sentences not included in the original training example. Box 3 shows how the rules match a new statement, and are applied to extract new leafShape and bladeDimension values.

This example illustrates a case where words are the basic unit of processing and the task is to classify words by using the context where they appear (*obovate* is identified as a leaf shape because it follows the phrase “leaf blade”).

In some applications, for example, named entity recognition (e.g., recognizing a word/phrase as a taxon name), an extraction target may appear in any context (e.g., a taxon name may be mentioned anywhere in a document). In these applications, the contextual information is less helpful in classifying a word/phrase than the letter combinations within the names. In NetiNeti, for example, a Naïve Baysian algorithm (a supervised learning algorithm based on Bayes conditional probability theorem) uses letter combinations to identify candidate taxon names [32]. When several training examples indicate names like *Turdus migratorius *are taxon names, NetiNeti may learn that a two-word phrase with the first letter capitalized and the last word ending with “*us*” (e.g.,* Felis catus*) is probably a taxon name, even though *Felis catus* has never appeared in training examples.

Supervised learning algorithms can be more difficult to use in biology largely because compiling large training datasets can be labor intensive, which decreases the adaptability and scalability of an algorithm to new document collections. Hundreds of controlled vocabularies exist for biological sciences, which can provide some training information to an algorithm but are often not comprehensive [16].

Unsupervised learning algorithms do not use training examples. These algorithms try to find hidden structure in unlabeled data using characteristics of the text itself. Well-known unsupervised learning algorithms include clustering algorithms, dimensionality reduction, and self-organization maps, to name a few. Cui et al. Boufford [14] designed an unsupervised algorithm to identify organ names and organ properties from morphological description sentences. The algorithm took advantage of a recurring pattern in which plural nouns that start a sentence are organs and a descriptive sentence starts with an organ name followed by a series of property descriptors. These characteristics of descriptive sentences allow an unsupervised algorithm to discover organ names and properties.

The procedure may be illustrated by using a set of five descriptive statements taken from Flora of North America (Box 4).

Because *roots* is a plural noun and starts statement 1 (in addition, the words *rooting* or *rooted* are not seen in the entire document collection, so *roots* is unlikely a verb) the algorithm infers *roots* is an organ name. Then, what follows it (i.e., *yellow*) must be a property. The algorithm remembers *yellow* is a property when it encounters statement 2 and it then infers that *petals* is an organ. Similarly, when it reads statement 3, because *petals* is an organ, the algorithm infers *absent* is a property, which enables the algorithm to further infer *subtending bracts* and *abaxial hastular* in statements 4 and 5 are organs. This example shows that by utilizing the description characteristics, the algorithm is able to learn that *roots*, *petals*, *subtending bracts*, and *abaxial hastular* are organ names and *yellow* and *absent* are properties, without using any training examples, dictionaries, or ontologies.

Because not all text possesses the characteristics required by the algorithm developed by Cui et al. [14], it cannot be directly applied to all taxon descriptions. However, because descriptions with those characteristics do exist in large numbers and because of the low overhead (in terms of preparing training examples) of the unsupervised learning algorithm, it is argued that unsupervised learning should be exploited when possible, such as when preparing text for a supervised learning task [16].

## 3. Review of Biodiversity Information Extraction Systems Annotation

 Our review describes the features of each system existing at the time of this writing. Many of the systems are being constantly developed with new features and enhanced capabilities. We encourage the readers to keep track of the development of these systems.

 Once text has been digitized, it can be annotated in preparation for an IE task or for use as training data for algorithms (Figure 2 number 8). Both aims require different levels of annotation granularity, which can be accomplished manually or automatically using annotation software. A low level of granularity (coarse) is helpful for identifying blocks of text useful for IE. As mentioned before, not all text is useful for every IE task. In the practice of systematics, taxonomists need text containing nomenclatural acts which may be discovered and annotated automatically through terms such as “sp. nov.” and “nov. comb.” Annotation of these text blocks is helpful for algorithms designed to extract information about species. A finer granularity is needed for training data annotation. Words or phrases within a taxonomic work may be annotated as a name, description, location, and so forth. High granularity is more helpful for training a machine-learning algorithm but imposes a larger cost in time needed to do the manual annotation. There must be a balance between level of granularity and amount of manual investment which is determined by the specific goals at hand.

Manual annotation is very time consuming but can be assisted with annotation software. Several software packages aid with this.

**taXMLit**

TaXMLit is an interface to allow annotation of taxonomic literature [51]. It was developed using botanical and zoological text, but also works well on paleontological text. This system is designed for annotation of text elements such as “description” and “locality.” This system requires a fairly large amount of human intervention and is not widely accepted.

**GoldenGATE**

GoldenGATE is an annotation tool for marking up taxonomic text in XML according to taxonX schema (http://plazi.org/files/GoldenGATE_V2_end_user_manual.pdf, [53]. Most of the annotation is done semi-automatically, with users checking the correctness of the annotations in the GoldenGATE editor that facilitates manual XML mark up. There are several plugins available for GoldenGATE, including modules for annotation specific to ZooTaxa and new plugins can be relatively easily added. The system is implemented in JAVA. This system performs best with text marked up with basic html tags (such as paragraph and header) and high-quality OCR.

**ABNER**

ABNER is an entity recognition algorithm designed specifically for the biomedical literature [54]. It uses a conditional random fields (CRF) model. This is a type of Bayesian statistics, wherein the computer uses characteristics of the text to determine the probability that a given term should be annotated as a given class. In this case, the available classes are: protein, DNA, RNA, Cell line, and Cell type. A human uses a point-and-click interface to confirm the algorithm results and add the annotation.

**OnTheFly**

OnTheFly is a text annotator that automatically finds and labels names of proteins, genes, and other small molecules in Microsoft Office, pdf, and text documents [55]. A user submits a file through the interface and it converts the file of interest into html and sends it to the Reflect tool. This tool looks for names and synonyms of proteins and small molecules to annotate as such [56]. It uses a list of 5.8 million molecule names from 373 organisms and returns matching terms. Clicking on an annotated term returns a pop-up window with additional information. In addition, this tool can create a graphical representation of the relationships between these entities using the STITCH database [57].

**Phenex**

Phenex was designed for use in the phenotypic literature [58]. It is a user interface that aids in manual annotation of biological text using terms from existing ontologies. Phenex allows users to annotate free text or NEXUS files. A core function of this software is to allow users to construct EQ (Entity:Quality) statements representing phenotypes. An EQ statement consists of two parts, a character (entity) and state (quality). The character is described using a term from an anatomy ontology and the state of that character is described using a term from a quality ontology (see, e.g., [59]). An example would be supraorbital bone:sigmoid. The fact that sigmoid is a shape is inferred from the PATO ontology and thus does not have to be specifically mentioned in the EQ statement (within [59] see Figure 1). Users can load the ontology containing the terms they want to use for annotation into Phenex which has an auto-complete function to facilitate work. The Phenex GUI provides components for editing, searching, and graphical displays of terms. This software is open source, released under the MIT license (http://phenoscape.org/wiki/Phenex).

### 3.1. Digitization

 The first step to making older biological literature machine readable is digitization (number 4 in Figure 2). Book pages can be scanned as images of text and made into pdf files, but cannot be submitted to NLP processing in this form. To make the text accessible, it must be OCRed (Optical Character Recognition) to translate the image of text (such as  .pdf) into actual text (such as  .txt). The Biodiversity Heritage Library is in the process of digitizing 600,000 pages of legacy text a month, making them available as pdf image files and OCR text files [45]. Most modern publications are available as pdf and html files from the publisher (and thus do not need to be scanned or OCRed). Images of text can be run through software designed to OCR files on desktop computers or as a web service (i.e., http://www.onlineocr.net/). OCR of handwriting is very different from that of text and can be quite difficult as there are as many handwriting styles as there are people. However, this type of OCR can be very important because significant portions of biodiversity data are only available as handwriting, such as museum specimen labels and laboratory notebooks. Algorithms do exist and are used for OCR of handwritten cities, states, and zip codes on envelopes and handwritten checks [46, 47].

 OCR is not a perfect technology. It is estimated that >35% of taxon names in BHL OCR files contain an error [45, 48, 49]. This is skewed, however, as older documents that use nonstandard fonts carry the majority of the errors [49]. Biodiversity literature can be especially difficult to OCR as they often have multiple languages on the same page (such as Latin descriptions), an expansive historical record going back to the 15th Century (print quality and consistency issues), and an irregular typeface or typesetting [48]. OCR is poor at distinguishing indentation patterns, bold, and italicized text, which can be important in biodiversity literature [50, 51]. The current rate of digitization prohibits manual correction of these errors. Proposed solutions include components of crowd-sourcing manual corrections and machine-learning for automated corrections [48].

OCR errors may be overcome by using “fuzzy” matching algorithms that can recognize the correct term from the misspelled version. TAXAMATCH is a fuzzy matching algorithm for use in taxonomy [52]. The need for a “fuzzy matching” algorithm for detection of similar names is apparent for functions such as search, federation of content, and correction of misspellings or OCR errors. TAXAMATCH is a tool that uses phonetic- and nonphonetic-based near-match algorithms that calculate the distance of the given letter combination to a target name included in a reference database [52]. A letter combination with a close proximity to a target name is proposed as a fuzzy match. This system is being successfully used to increase hits in species databases [52] and is optimized for human typos rather than OCR errors. The php version of this code is available through Google code (http://code.google.com/p/taxamatch-webservice/) and a Ruby version is available through git hub (https://github.com/GlobalNamesArchitecture/taxamatch_rb).

### 3.2. Names Recognition and Discovery

 A taxonomic name is connected to almost every piece of information about an organism, making names near universal metadata in biology (see Rod Page's iphylo blog entry http://iphylo.blogspot.com/2011/04/dark-taxa-genbank-in-post-taxonomic.html for an exception). This can be exploited to find and manage nearly all biological data. No life-wide, comprehensive list of taxonomic names exists, but the Global Names Index (GNI) holds 20 million names and NameBank (http://www.ubio.org/index.php?pagename=namebank) holds 10 million names. There are also exclusive lists of taxonomically creditable names such as the Catalogue of Life (CoLP) and the Interim Register of Marine and Non-marine Genera (IRMNG). These lists hold 1.3 million and 1.6 million names, respectively.

Taxonomic names discovery (or Named Entity Recognition in computer science parlance) can be achieved through several approaches. *Dictionary-based approaches *rely on an existing list of names. These systems try to find names on the list directly in the text. The major drawback of this approach in biology is that there is no comprehensive list of names and terms including all misspellings, variants, and abbreviations. Dictionary-based approaches can also miss synonyms and ambiguous names. Some algorithms have been developed to aid dictionary-based approaches with recognizing variants of names in the list (e.g., see algorithms described below). *Rule-based approaches* work by applying a fixed set of rules to a text. This approach is capable of dealing with variations in word order and sentence structure in addition to word morphology. The major drawback is that the rule sets are handmade (and, therefore, labor intensive) and are rarely applicable to multiple domains. *Machine*-*learning approaches* use rule sets generated by the machine using statistical procedures (such as Hidden Markov Models). In this approach, algorithms are trained on an annotated body of text in which names are tagged by hand. The algorithms can be applied to text in any discipline as long as appropriate training data are available. All of these approaches have strengths and weaknesses, so they are often combined in final products.

Several algorithms have been developed that are capable of identifying and discovering known and unknown (to the algorithm) taxon names in free text. These are discussed below and their performance metrics are given in Table 4.

**TaxonGrab**

TaxonGrab identifies names by using a combination of nomenclatural rules and a list (dictionary) of non-taxonomic English terms [31]. As most taxonomic names do not match words in common parlance, the dictionary can be used as a “black list” to exclude terms. This is not always the case because some Latin names match vernacular names, such as bison and *Bison bison*. The algorithm scans text for terms that are not found in the black list. It treats these as candidate names. These terms are then compared to the capitalization rules of Linnaean nomenclature. Algorithms of this type have low precision because misspelled, non-English words, medical, or legal terms would be flagged as a candidate name. However, these terms can be iteratively added to the black list, improving future precision. This method does have the advantage of not requiring a complete list of species names, but can only be used on English texts. Later, several additional rules were added to create a new product, FAT [60]. FAT employs “fuzzy” matching and structural rules sequentially so that each rule can use the results of the last. The TaxonGrab code is available at SourceForge, but the FAT code is not. FAT is a part of the plazi.org toolset for markup of taxonomic text.

**TaxonFinder**

TaxonFinder identifies scientific names in free text by comparing the name to several lists embedded into the source code ([61], Leary personal comments). These lists are derived from a manually curated version of NameBank (http://www.ubio.org/index.php?pagename=namebank). A list of ambiguous names was compiled from words that are names, but are more often used in common parlance, like pluto or tumor. TaxonFinder breaks documents into words and compares them to the lists individually. When it encounters a capitalized word, it checks the “genus” and “above-genus” name lists. If the word is in the above-genus list, but not in the ambiguous name list, it is returned as a name. If it is in the genus list, the next word is checked to see if it is in lower case or all caps and to see if it is in the “species-or-below” name list. If it is, then the process is repeated with the next word until a complete polynomial is returned. If the next word is not in the list, then the previous name is returned as a genus. TaxonFinder is limited to dictionaries and thus will not find new names or misspellings but can discover new combinations of known names. This system can have both high precision and recall with a higher score in precision (more false negatives than false positives). A previous version of TaxonFinder, FindIT (http://www.ubio.org/tools/recognize.php), had the ability to identify authorship by recognizing the reference (usually a taxonomist's name), which TaxonFinder does not do (http://code.google.com/p/taxon-name-processing/wiki/nameRecognition). A new, Apache Lucene-based name indexer is now available from GBIF which is based on TaxonFinder (http://tools.gbif.org/namefinder/). The source code for TaxonFinder is available at Google code (http://code.google.com/p/taxon-finder/).

**NetiNeti**

NetiNeti takes a more unsupervised approach to names extraction [32]. The system uses natural language processing techniques involving probabilistic classifiers (Naive Bayes classifier by default) to recognize scientific names in an arbitrary document. The classifier is trained to recognize characteristics of scientific names as well as the context. The algorithm uses “white list” and “black list” detection techniques in a secondary role. As a result, scientific names not mentioned in a white list or names with OCR errors or misspellings are found with great accuracy. Some of the limitations of NetiNeti include an inability to identify genus names less than four letters long, the assumption of one letter abbreviations of genera, and limitation of contextual information available to one word on either side of a candidate name. The code of this tool is written in Python and is going to be released under GPL2 license at https://github.com/mbl-cli/NetiNeti.

**Linnaeus**

This is a list-based system designed specifically for identifying taxonomic names in biomedical literature and linking those names to database identifiers [33]. The system recognizes names contained in a white list (based on the NCBI classification and a custom set of synonyms) and resolves them to an unambiguous NCBI taxonomy identifier within the NCBI taxonomy database (http://www.ncbi.nlm.nih.gov/books/NBK21100/). In this way, multiple names for one species are normalized to a single identifier. This system is capable of recognizing and normalizing ambiguous mentions, such as abbreviations (*C. elegans*, which refers to 41 species) and acronyms (CMV, which refers to 2 species). Acronyms that are not listed within the NCBI classification are discovered using the Acromine service [62] and a novel acronym detector built into LINNAEUS that can detect acronym definitions within text (in the form of “species (acronym)”). Ambiguous mentions that are not resolvable are assigned a probability of how likely the mention refers to a species based on the relative frequency of nonambiguous mentions across all of MEDLINE. Applying a black list of species names that occur commonly in the English language when not referring to species (such as the common name spot) greatly reduces false positives. LINNAEUS can process files in XML and txt formats and give output in tab-separated files, XML, HTML and MySQL database tables. This code is available at SourceForge (http://sourceforge.net/projects/linnaeus/).

**OrganismTagger**

This system uses the NCBI taxonomy database (http://www.ncbi.nlm.nih.gov/books/NBK21100/) to generate semantically enabled lists and ontology components for organism name extraction from free text [34]. These components are connected to form a work flow pipeline using GATE (the General Architecture for Text Engineering; [63, 64]). These components are a combination of rule-based and machine-learning approaches to discover and extract names from text, including strain designations. To identify strains not in the NCBI taxonomy database, OrganismTagger uses a “strain classifier,” a machine-learning (SVM model) approach trained on manually annotated documents. After the strain classifier is applied, organism names are first detected, then normalized to a single canonical name and grounded to a specific NCBI database ID. The semantic nature of this tool allows it to output data in many different formats (XML, OWL, etc.). This code along with supporting materials is available under an open source license at http://www.semanticsoftware.info/organism-tagger.

### 3.3. Morphological Character Extraction

Morphological characters of organisms are of interest to systematists, evolutionary biologists, ecologists, and the general public. The examples used in Figure 4 are typical of morphological descriptions. The kinds of language used in biodiversity science has the following characteristics that make it difficult for general-purpose parsers to process [15, 65, 66].

1. Specialized Language. Most scientific terms are not in the lexicons of existing parsers. Even if they were, biological terms are more ambiguous than general English [67]. General English has 0.57% ambiguous terms while gene names have 14.2% ambiguity. Taxonomic homonyms are 15% at the genus level (http://www.obis.org.au/irmng/irmng_faq/). Life Science literature also relies heavily on abbreviations [68]. There were over 64,000 new abbreviations introduced in 2004 in the biomedical literature alone and an average of one new abbreviation every 5–10 abstracts [69]. Dictionaries, such as the Dictionary of Medical Acronyms and Abbreviations can help, but most dictionaries contain 4,000 to 32,000 terms, which is only a fraction of the estimated 800,000 believed to exist [69, 70]. This means that dictionary-based approaches will not scale to work in biology.
2. Diversity. Descriptions are very diverse across taxon groups. Even in one group, for example, plants, variations are large. Lydon et al. [71] compared and contrasted the descriptions of five common species in six different English language Floras and found the same information in all sources only 9% of the time. They also noted differences in terminology usage across Floras.
3. Syntax differences. Many species descriptions are in telegraphic sublanguage (that lacks of verbs) but there are also many descriptions conforming to more standard English syntax. Parsers expecting standard English syntax often mistake other groups of words for verbs when parsing telegraphic sublanguage because they expect to see verbs in a sentence. There is not typically standardized syntax across different taxon groups or even within the same group.

Taylor [15, 72] manually constructed a grammar and a lexicon of 2000 characters, and character states (1500 from Radford [73] and 500 from descriptive text) to parse the Flora of New South Wales (4 volumes) and volume 19 of the Flora of Australia. The goal of parsing these Floras was to create sets of organism part, character, and character state from each description. These statements can be extracted from morphological taxon descriptions using the hand-crafted parser to get a machine-readable set of facts about organism characteristics. While the sublanguage nature of the plant descriptions used by Taylor [15, 72] made it easier to construct the grammar and lexicon manually, the author acknowledged the limited coverage they could be expected to achieve (60–80% recall was estimated based on manual examination of output). Algorithms for machine-aided expansion of the lexicon were suggested; however, at the time automated creation of rules was believed to be too difficult.

Since Taylor [15, 72], a variety of methods have been used to extract morphological traits from morphological descriptions. Their performance metrics are given in Table 4.

**X-Tract**

X-tract [35] was an interactive tool to extract morphological information from Flora of North America (FNA) descriptions available as a print and HTML version. X-tract used HTML tags embedded in the FNA pages to identify the morphological description sections. It used a glossary to classify each word in a description as structure (i.e., organs or part of organs) or character states. If a word was a character state, its corresponding characters were looked up in the glossary. Then, X-tract created a form to display the structures, substructures, characters, and character states extracted from a document for a user to review, modify, and save to a database. Evaluation of the extraction accuracy or the extent of user intervention was not provided.

**Worldwide Botanical Knowledge Base**

Jean-Marc Vanel initiated a project called Worldwide Botanical Knowledge Base, which also takes the approach of parsing plus glossary/lexicon. It marks up morphological descriptions at sentence level (e.g., leaf blade obovate is marked as “leaf blade”) without extracting detailed character information. It stores extracted information in XML files instead of a relational database as Taylor [15, 72] and Abascal and Sánchez [35]. The project aims to support queries on species descriptions in botanical databases. The database search seems to have stopped working (http://jmvanel.free.fr/protea.html). The parser was reported to work on Flora of China and it can be downloaded from the website (http://wwbota.free.fr/). However, as of the time of this publication, the authors were unable to use the parser.

**Terminator**

Diederich, Fortuner and Milton [74] developed a system called Terminator, which used a hand-crafted plant glossary that amounts to an ontology including structure names, characters and character states to support character extraction. The extraction process was a combination of fuzzy keyword match and heuristic extraction rules. Because Terminator was an interactive system (i.e., a human operator selects correct extractions), the evaluation was done on 16 descriptions to report the time taken to process them. Extraction performance was evaluated only on 1 random sample: for non-numerical characters, 55% of the time a perfect structure/character/value combination was among the first 5 candidates suggested by the system.

**MultiFlora**

Similar to previous works, Wood, Lydon, and colleagues' MultiFlora project (http://intranet.cs.man.ac.uk/ai/public/MultiFlora/MF1.html) started with manual analysis of description documents. They created an ontology manually, which included classes of organs (i.e., petal) and features (i.e., yellow) linked by properties (i.e., hasColor). They also manually created a gazetteer, which included terms referring to the organs and features that served as a lookup list. The prototype MultiFlora system used a combination of keyword matching, internal and contextual pattern matching, and shallow parsing techniques provided by GATE to extract organ and feature information from a small collection of morphological descriptions (18 species descriptions, recall, and precision were in the range of mid 60% to mid 70%; [66, 75]). While the work of Wood, Lydon, and colleagues shows that using descriptions from different sources can be used to improve recall, the authors acknowledged that organs not included in the manually-created gazetteer/ontology have to be marked as “unknown.” The extraction results were output in RDF triples and used to build a knowledgebase about plants, which is not related to Worldwide Botanical Knowledge Base reviewed earlier. RDF is a type of programming language that allows a user to make machine readable assertions in the form of an RDF triple. The EQ format mentioned earlier is a similar format used in biology. The advantage to using ontology-supported RDF/EQ is that multiple data providers can use the same ontological identifier for the same term. In this way, statements become machine-readable and can be linked regardless of the source. With ontological support, machine-based logic reasoning has become possible. An immediate application of this type of reasoning and a pool of RDF triples describing species morphology is a specimen identification key. RDF is supported by more recent biodiversity IE systems as an output format.

**MARTT**

MARTT [76] is an automated description markup system employing a supervised machine-learning algorithm. The system marks up a description sentence-by-sentence with tags that indicate the subject, for example, “stem” is tagged in the text statement “stem solitary.” MARTT along with a test collection is downloadable from http://sites.google.com/site/biosemanticsproject/project-progress-wiki. Wei [77] conducted an exploratory study of the application of information fusion techniques to taxonomic descriptions. It confirmed Wood et al. [75] finding that combining multiple descriptions of the same species from different sources and different taxonomic ranks can provide the researchers more complete information than any single description. Wei used MARTT [76] and a set of heuristic rules to extract character information from descriptions of taxa published in both FNA and Flora of China (FoC) and categorized the extracted information between the two sources as either identical, equivalent, subsumption, complementary, overlap, or conflict. Non-conflict information from both sources was then merged together. The evaluation was conducted involving 13 human curators verifying results generated from 153 leaf descriptions. The results show that the precisions for genus level fusion, species level fusion, FNA genus-species fusion, and FoC genus-species fusion were 77%, 63%, 66%, and 71%, respectively. The research also identified the key factors that contribute to the performance of the system: the quality of the dictionary (or the domain knowledge), the variance of the vocabulary, and the quality of prior IE steps.

**WHISK**

Tang and Heidorn [13] adapted WHISK [78] to extract morphological character and other information from several volumes of FNA to show that IE helps the information retrieval system SEARFA (e.g., retrieval of relevant documents). The “pattern matching” learning method used by WHISK is described in Section 2. The pattern matching algorithm was assisted by a knowledge base created by manually collecting structure and character terms from training examples. The IE system was evaluated on a relatively small subset of FNA documents and it was evaluated on different template slots (see Table 1 for examples of template slots) separately. Different numbers of training and/or test examples were used for different slots (training examples ranged from 7 to 206, test examples ranged from 6 to 192) and the performance scores were obtained from one run (as opposed to using the typical protocol for supervised learning algorithms). The system performed perfectly on nonmorphological character slots (Genus, Species, and Distribution). The recall on morphological character slots (Leaf shape, Leaf margin, Leaf apex, Leaf base, Leaf arrangement, Blade dimension, Leaf color, and Fruit/nut shape) ranged from 33.33% to 79.65%. The precision ranged from 75.52% to 100%. Investigation of human user performance on plant identification using internet-based information retrieval systems showed that even with imperfect extraction performance, users were able to make significantly more identifications using the information retrieval system supported by the extracted character information than using a keyword-based full-text search system.

**CharaParser**

All IE systems reviewed above relied on manually created vocabulary resources, whether they are called lexicons, gazetteers, or knowledge bases. Vocabularies are a fundamental resource on which more advanced syntactic and semantic analyses are built. While manually collecting terms for a proof-of-concept system is feasible, the manual approach cannot be scaled to the problem of extracting morphological traits of all taxa. Cui, Seldon & Boufford [14] proposed an unsupervised bootstrapping based algorithm (described in Section 2) that can extract 93% of anatomical terms and over 50% character terms from text descriptions without any training examples. This efficient tool may be used to build vocabulary resources that are required to use various IE systems on new document collections.

**Figure 5:**
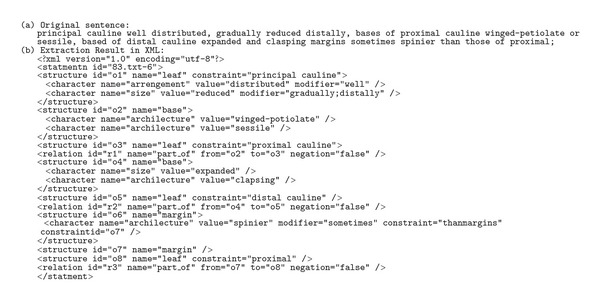
Extraction result from a descriptive sentence.

This unsupervised algorithm has been used in two IE systems [36, 79]. One of the systems used intuitive heuristic rules to associate extracted character information with appropriate anatomical structures. The other system (called CharaParser) adapted a general-purpose syntactic parser (Stanford Parser) to guide the extraction. In addition to structures and character extraction, both systems extract constraints, modifiers, and relations among anatomical structures (e.g., head *subtended by* distal leaves; pappi *consist of* bristles) as stated in a description. Both systems were tested on two sets of descriptions from volume 19 of FNA and Part H of Treatise on Invertebrate Paleontology (TIP); each set consisted of over 400 descriptions. The heuristic rule-based system achieved precision/recall of 63%/60% on the FNA evaluation set and 52%/43% on the TIP evaluation set on character extraction. CharaParser performed significantly better and achieved precision/recall of 91%/90% on the FNA set and 80%/87% on the TIP set. Similar to Wood et al. [66], Cui and team found the information structure of morphological descriptions was too complicated to be represented in a typical IE template (such as Table 1). Wood et al. [66] designed an ontology to hold the extracted information, while Cui and team used XML to store extracted information (Figure 5). CharaParser is expected to be released as an open-source software in Fall 2012. Interested readers may contact the team to obtain a trial version before its release.

### 3.4. Integrated IE Systems

 Tang and Heidorn [13] supervised learning IE system, MutiFlora, and the CharaParser system, all reviewed before, can be described using the reference model depicted in Figure 2. Here, we describe another system that integrates formal ontologies. This is the text mining system that is currently under development by the Phenoscape project (http://www.phenoscape.org/). The goal of Phenoscape is to turn text phenotype descriptions to EQ expressions [80] to support machine reasoning of scientific knowledge as a transforming way of conducting biological research. In this application, EQ expressions may be considered both the IE template and a data standard. The input to the Phenoscape text mining system is digital or OCRed phylogenetic publications. The character descriptions are targeted (1 character description = 1 character statement + multiple character state statements) and used to form the taxon-character matrix. The target sections are extracted by student assistants using Phenex and put into NeXML (http://www.nexml.org/) format. NeXML is an exchange standard for representing phyloinformatic data. It is inspired by the commonly used NEXUS format, but more robust and easier to process. There is one NeXML file for a source text. NeXML files are the input to CharaParser, which performs bootstrapping-based learning (i.e., unsupervised learning) and deep parsing to extract information and output candidate EQ expressions. CharaParser learns lexicons of anatomy terms and character terms from description collections. Learned terms are reviewed by biologist curators (many OCR errors are detected during this step). Terms that are not in existing anatomy ontologies are proposed to the ontologies for addition. The lexicons and ontologies are the knowledge entities that the text mining system iteratively uses and enhances. With new terms added to the ontologies, the system replaces the terms in candidate EQ statements with term IDs from the ontologies. For example, [E]tooth [Q]large is turned into [E]TAO: 0001625 [Q]PATO: 0001202. The candidate EQ expressions are reviewed and accepted by biologist curators using Phenex. Final EQ expressions are loaded into the Phenoscape Knowledge base at http://kb.phenoscape.org/. This EQ populated knowledge base supports formal logical reasoning. At the time of writing, the developing work is ongoing to integrate CharaParser with Phenex to produce an integrated text-mining system for Phenoscape. It is important to notice that the applicability of Phenex and CharaParser is not taxon specific.

## 4. Conclusion

 NLP approaches are capable of extracting large amounts of information from free text. However, biology text presents a unique challenge (compared to news articles) to machine-learning algorithms due to its ambiguity, diversity, and specialized language. Successful IE strategies in biodiversity science take advantage of the Linnaean binomial structure of names and the structured nature of taxon descriptions. Multiple tools currently exist for fuzzy matching of terms, automated annotation, named-entity recognition, and morphological character extraction that use a variety of approaches. None have yet been used on a large scale to extract information about all life, but several, such as CharaParser, show potential to be used in this way. Further improvement of biodiversity IE tools could be achieved through increased participation in the annual BioCreative competitions (http://www.biocreative.org/) and assessing tool performance on publicly available document sets so that comparison between systems (and thus identification of methods that have real potential to address biodiversity IE problems) becomes easier.

 A long-term vision for the purpose of making biodiversity data machine readable is the compilation of semantic species descriptions that can be linked into a semantic web for biology. An example of semantic species information can be found at TaxonConcept.org. This concept raises many questions concerning semantics which are outside the scope of this paper, such as what makes a “good” semantic description of a species. Many of these issues are technical and are being addressed within the computer science community. There are two data pathways that need to be developed to achieve the semantic web for biology. One is a path going forward, in which new data are made machine-readable from the beginning of a research project. The model of mobilizing data many years after collection with little to no data management planning during collection is not sustainable or desirable going into the future. Research is being applied to this area and publishers, such as Pensoft, are working to capture machine-readable data about species at the point of publication. The other is a path for mobilizing data that have already been collected. NLP holds much promise in helping with the second path.

Mobilizing the entirety of biodiversity knowledge collected over the past 250 years is an ambitious goal that requires meeting several challenges from both the taxonomic and technological fronts. Considering the constantly changing nature of biodiversity science and the constraints of NLP algorithms, best results may be achieved by drawing information from high quality modern reviews of taxonomic groups rather than repositories of original descriptions. However, such works can be rare or nonexistent for some taxa. Thus, issues such as proper aggregation of information extracted from multiple sources on a single subject (as mentioned above) still need to be addressed. In addition, demanding that a modern review be available somewhat defeats the purpose of applying NLP to biodiversity science. While using a modern review may be ideal when available, it should not be required for information extraction.

 Biodiversity science, as a discipline, is being asked to address numerous challenges related to climate change, biodiversity loss, and invasive species. Solutions to these problems require discovery and aggregation of data from the entire pool of biological knowledge including what is contained exclusively in print holdings. Digitization and IE on this scale is unprecedented. Unsupervised algorithms hold the greatest promise for achieving the scalability required because they do not require manually generated training data. However, most successful IE algorithms use combinations of supervised and unsupervised strategies and multiple NLP approaches because not all problems can be solved with an unsupervised algorithm. If the challenge is not met, irreplaceable data from centuries of research funded by billions of dollars may be lost. The annotation and extraction algorithms mentioned in this manuscript are key steps toward liberating existing biological data and even serve as preliminary evidence that this goal can be achieved.

## Figures and Tables

**Box 1:**
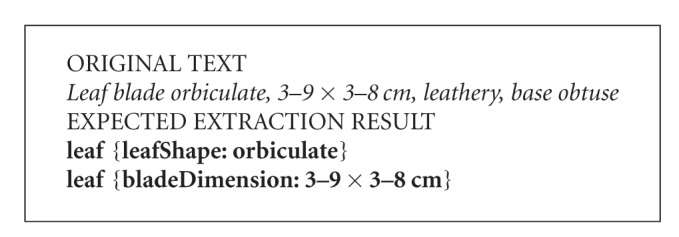


**Box 2:**
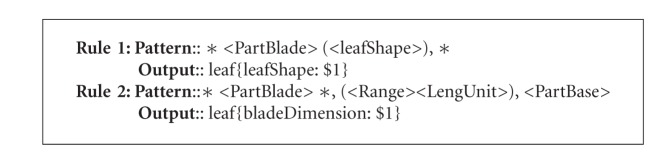


**Box 3:**
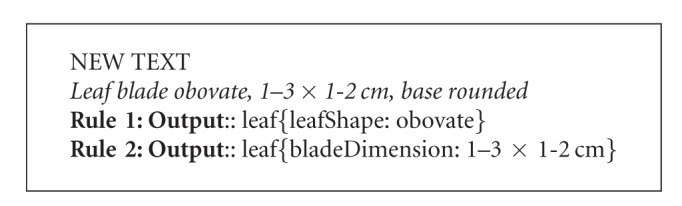


**Box 4:**
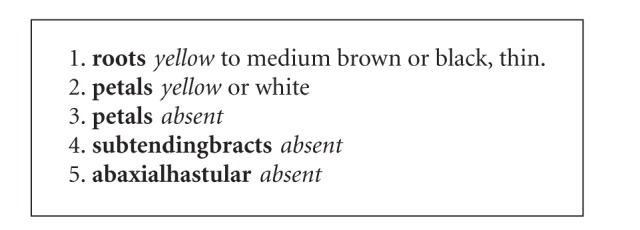


**Table 1:** From Tang and Heidorn [13]. An example template for morphological character extraction.

Template slots	Extracted information
Genus	Pellaea
Species	mucronata
Distribution	Nev. Calif.
Leaf shape	ovate-deltate
Leaf margin	dentate
Leaf apex	mucronate
Leaf base	
Leaf arrangement	clustered
Blade dimension	
Leaf color	
Fruit/nut shape	

**Table 2:** Information extraction tasks outlined by the MUCs and their descriptions.

Task	Description
Named entity	Extracts names of entities
Coreference	Links references to the same entity
Template element	Extracts descriptors of entities
Template rotation	Extracts relationships between entities
Scenario template	Extracts events

**Table 3:** Existing IE systems for biology [17–26].

System	Approach	Structure of Text	Knowledge in	Application domain	Reference
AkanePPI	shallow parsing	sentence-split, tokenized, and annotated		protein interactions	[17]
EMPathIE	pattern matching	text	EMP database	enzymes	[18]
PASTA	pattern matching	text	biological lexicons	protein structure	[19]
BioIE	pattern matching	xml	dictionary of terms	biomedicine	[20]
BioRAT	pattern matching, sub-language driven	could be xml, html, text or asn.1, can do full-length pdf papers (converts to text)	dictionary for protein and gene names, dictionary for interactions, and synonyms; text pattern template	biomedicine	[21]
Chilibot	shallow parsing	not sure what was used in paper, but could be xml, html, text or asn.1	nomenclature dictionary	biomedicine	[22]
Dragon Toolkit	mixed syntactic semantic	text	domain ontologies	genomics	[23]
EBIMed	pattern matching	xml	dictionary of terms	biomedicine	[24]
iProLINK	shallow parsing	text	protein name dictionary, ontology, and annotated corpora	proteins	[25]
LitMiner	mixed syntactic semantic	web documents		Drosophila research	[26]

**Table 4:** Performance metrics for the names recognition and morphological character extraction algorithms reviewed. Recall and precision values may not be directly comparable between the different algorithms. NA: not available [30].

Tool	Recall	Precision	Test Corpora	Reference
TaxonGrab	>94%	>96%	Vol. 1 Birds of the Belgian Congo by Chapin	[31]
FAT	40.2%	84.0%	American Seashells by Abbott	[32]
Taxon Finder	54.3%	97.5%	American Seashells by Abbott	[32]
Neti Neti	70.5%	98.9%	American Seashells by Abbott	[32]
LINNAEUS	94.3%	97.1%	LINNAEUS gold standard data set	[33]
Organism Tagger	94.0%	95.0%	LINNAEUS gold standard data set	[34]
X-tract	NA	NA	Flora of North America	[35]
Worldwide Botanical Knowledge Base	NA	NA	Flora of China	http://wwbota.free.fr/
Terminator	NA	NA	16 nematode descriptions	http://www.math.ucdavis.edu/~milton/genisys/terminator.html
MultiFlora	mid 60%	mid 70%	Descriptions of Ranunculus spp. from six Floras	http://intranet.cs.man.ac.uk/ai/public/MultiFlora/MF1.html
MARTT	98.0%	58.0%	Flora of North America and Flora of China	[30]
WHISK	33.33% to 79.65%	72.52% to 100%	Flora of North America	[13]
CharaParser	90.0%	91.0%	Flora of North America	[36]
